# High-Fat Diet-Induced Insulin Resistance in Single Skeletal Muscle Fibers is Fiber Type Selective

**DOI:** 10.1038/s41598-017-12682-z

**Published:** 2017-10-20

**Authors:** Mark W. Pataky, Haiyan Wang, Carmen S. Yu, Edward B. Arias, Robert J. Ploutz-Snyder, Xiaohua Zheng, Gregory D. Cartee

**Affiliations:** 1 Muscle Biology Laboratory, School of Kinesiology, University of Michigan, Ann Arbor, MI USA; 2 Applied Biostatistics Laboratory, University of Michigan, Ann Arbor, MI USA; 3 Department of Molecular and Integrative Physiology, University of Michigan, Ann Arbor, MI USA; 4 Institute of Gerontology, University of Michigan, Ann Arbor, MI USA

## Abstract

Skeletal muscle is the major site for insulin-stimulated glucose disposal, and muscle insulin resistance confers many negative health outcomes. Muscle is composed of multiple fiber types, and conventional analysis of whole muscles cannot elucidate fiber type differences at the cellular level. Previous research demonstrated that a brief (two weeks) high fat diet (HFD) caused insulin resistance in rat skeletal muscle. The primary aim of this study was to determine in rat skeletal muscle the influence of a brief (two weeks) HFD on glucose uptake (GU) ± insulin in single fibers that were also characterized for fiber type. Epitrochlearis muscles were incubated with [^3^H]-2-deoxyglucose (2DG) ± 100 µU/ml insulin. Fiber type (myosin heavy chain expression) and 2DG accumulation were measured in whole muscles and single fibers. Although fiber type composition of whole muscles did not differ between diet groups, GU of insulin-stimulated whole muscles from LFD rats significantly exceeded HFD values (P < 0.005). For HFD versus LFD rats, GU of insulin-stimulated single fibers was significantly (P < 0.05) lower for IIA, IIAX, IIBX, IIB, and approached significance for IIX (P = 0.100), but not type I (P = 0.776) fibers. These results revealed HFD-induced insulin resistance was attributable to fiber type selective insulin resistance and independent of altered fiber type composition.

## Introduction

Skeletal muscle is the major site for insulin-stimulated glucose disposal^1^, and skeletal muscle insulin resistance is a primary and essential event in the progression to type 2 diabetes^2^. Even in the absence of type 2 diabetes, insulin resistance confers negative health outcomes^3^. It is important to understand the processes responsible for insulin resistance of the skeletal muscle to develop interventions that effectively combat this health-related functional deficit.

Insulin resistance, concomitant with substantial weight gain and obesity, can be induced by feeding rodents a high fat diet (HFD) for a period of many weeks or months^4–7^. However, insulin resistance is detectable in rodents after only one to three weeks of a HFD, prior to major increases in body mass or body fat^8,9^. To gain insights about this rapid HFD-induced insulin resistance in skeletal muscle, we studied rats eating a two week HFD protocol that was previously reported to produce skeletal muscle insulin resistance^9^.

Fully understanding insulin resistance in skeletal muscle at the cellular level is challenging because this tissue includes multiple muscle fiber types that vary greatly in their metabolic properties^10^. The gold standard for muscle fiber type classification is based on myosin heavy chain (MHC) isoform expression, and type I, IIA, IIX, and IIB are the major MHC isoforms expressed in adult rat skeletal muscle^11^. The usual approach for assessing fiber type differences involves using muscle tissue (either whole muscles or muscle regions) that is enriched with a particular fiber type. Relatively few studies have considered a possible relationship between muscle fiber type and HFD-induced insulin resistance. The results of some, but not all of these earlier studies suggest that HFD effects on insulin-stimulated glucose uptake can differ between muscles with different fiber type composition^12–16^.

There are significant caveats in delineating fiber type differences based on conventional whole muscle analysis, including that: (1) no muscle is entirely composed of a single fiber type, (2) to our knowledge no rat muscle is primarily composed of type IIX fibers, (3) it is impossible to adequately evaluate fibers that express multiple MHC isoforms (known as hybrid fibers) using conventional whole muscle analysis, and (4) in addition to myocytes, muscle tissue contains many cell types, including vascular, adipose and neural cells. Because of these limitations, our laboratory has developed and validated a unique method for measuring both fiber type by MHC and glucose uptake in a single muscle fiber^17^. This approach has enabled the elucidation of fiber type-specific glucose uptake responses to various physiological conditions such as acute exercise, aging, and obesity^17–19^.

This study’s primary aim was to determine in rat skeletal muscle the influence of a short-term (2 weeks) HFD on glucose uptake ± insulin in single fibers that were also characterized for fiber type (types I, IIA, IIB, IIX, IIAX and IIBX). In addition, because some studies that compared rodents consuming a HFD for four or more weeks with healthy controls eating a low fat diet (LFD) have identified differences between the diet groups in their muscle fiber type composition^20–23^, a secondary aim was to determine in rat skeletal muscle the influence of short-term HFD on the fiber type composition as assessed by MHC isoform expression.

Insulin-stimulated glucose uptake by skeletal muscle relies on the expression of the insulin regulated GLUT4 glucose transporter protein^24,25^. Although we previously observed whole epitrochlearis muscle GLUT4 abundance was not altered by 2-weeks of HFD^9^, it seemed possible that the HFD might induce fiber type-selective changes in GLUT4 abundance. Therefore, our third aim was to assess the HFD effect on GLUT4 abundance in single fibers of differing fiber types as a potential mechanism for HFD-induced insulin resistance.

There is extensive interest in the possibility of a mitochondrial role in skeletal muscle insulin resistance^26–29^. However, prior studies have not determined the influence of HFD on both insulin-stimulated glucose uptake and the abundance of mitochondrial proteins in a fiber type selective manner at the single fiber level. Accordingly, our fourth aim was to assess in single fibers that had been characterized for fiber type the HFD effect on the abundance of six mitochondrial proteins that are functionally important in the electron transport chain and oxidative phosphorylation.

## Results

Following the diet intervention, the HFD animals compared to the LFD animals had a significantly greater estimated 2-week caloric intake (1883 ± 122 vs. 1624 ± 85 kcals; P < 0.005), body mass (326 ± 17 vs. 302 ± 11 g; P < 0.05), epididymal fat mass (6780 ± 942 vs. 4310 ± 441 mg; P < 0.001), and epididymal fat mass to body mass ratio (20 ± 2 vs. 14 ± 1 mg/g; P < 0.001).

**Figure 1:**
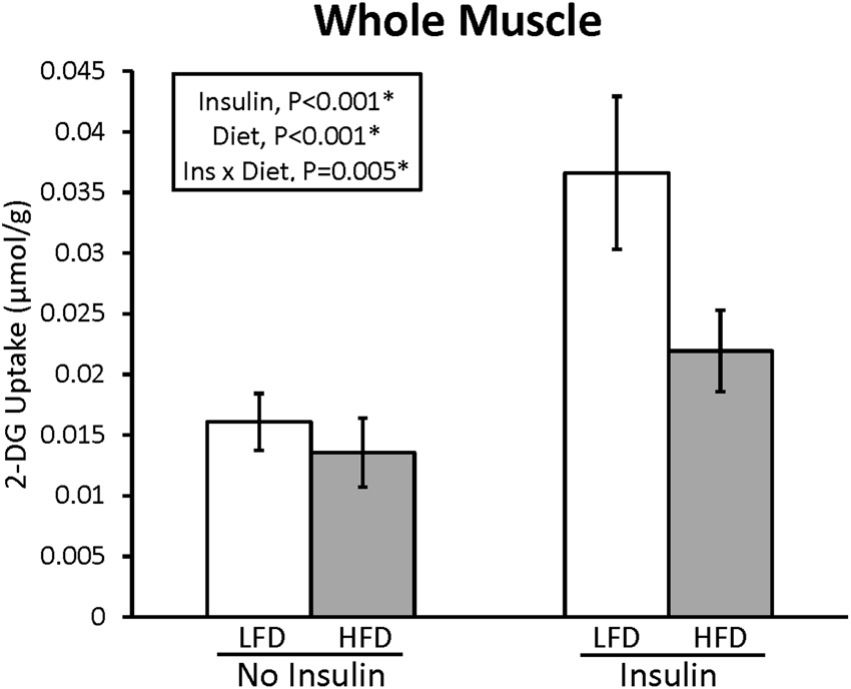
Effect of high fat diet (HFD) versus low fat diet (LFD) on 2-DG uptake in whole epitrochlearis muscles. P-values are displayed, and *P < 0.05 was considered statistically significant. Values are means ± 95% confidence interval.

For whole muscles, the LFD and HFD groups were not significantly different for the relative abundance of any of the MHC isoforms (Table 1 and Supplementary Figure 1). For whole muscles there was a significant (P < 0.010, n = 10 per group) interaction between diet and insulin for glucose uptake (Fig. 1). Post-hoc analysis indicated that insulin-independent glucose uptake was not significantly different between LFD and HFD groups, but glucose uptake of insulin-stimulated muscles was greater (P < 0.01) for LFD compared to HFD animals. Additionally, glucose uptake with insulin exceeded (P < 0.01) values with no insulin in each diet group.

**Table 1:** Relative myosin heavy chain isoform composition of whole epitrochlearis muscle.

% MHC I		% MHC IIA		% MHC IIX		% MHC IIB	
LFD	HFD	LFD	HFD	LFD	HFD	LFD	HFD
8.6 ± 1.8	7.5 ± 1.7	16.8 ± 2.3	15.0 ± 2.1	27.1 ± 3.6	26.2 ± 3.3	47.4 ± 5.5	51.3 ± 4.2

Myosin heavy chain (MHC) isoforms of whole epitrochlearis muscles from rats in the low fat diet (LFD) and high fat diet (HFD) groups were separated by SDS-PAGE, and the gels were stained with SimplyBlue^TM^ SafeStain. Resulting bands were quantified by densitometry and expressed as relative values (%). Values are means ± 95% confidence interval.

MHC isoform of each single fiber was identified by SDS PAGE with subsequent protein staining, and six distinct fiber types were identified: type I, IIA, IIAX, IIX, IIBX, and IIB (Supplementary Figures 2 and 3). For single fibers in the LFD group, the total number of fibers (no insulin/insulin) isolated for each fiber type were: type I (18/28), type IIA (129/175), type IIAX (32/46), type IIX (142/102), type IIBX (73/49), and type IIB (114/116). The number of muscles (no insulin/insulin) from which the LFD fibers were isolated were: type I (3/7), type IIA (10/10), type IIAX (8/9), type IIX (10/9), type IIBX (10/10), type IIB (10/10). In the HFD group, the total number of fibers (no insulin/insulin) isolated for each fiber type were: type I (10/12), type IIA (153/187), type IIAX (32/18), type IIX (127/139), type IIBX (67/80), and type IIB (123/100). The number of muscles (no insulin/insulin) from which the HFD fibers were isolated were: type I (3/4), type IIA (8/10), type IIAX (6/5), type IIX (10/10), type IIBX (9/10), type IIB (10/10).

**Figure 2:**
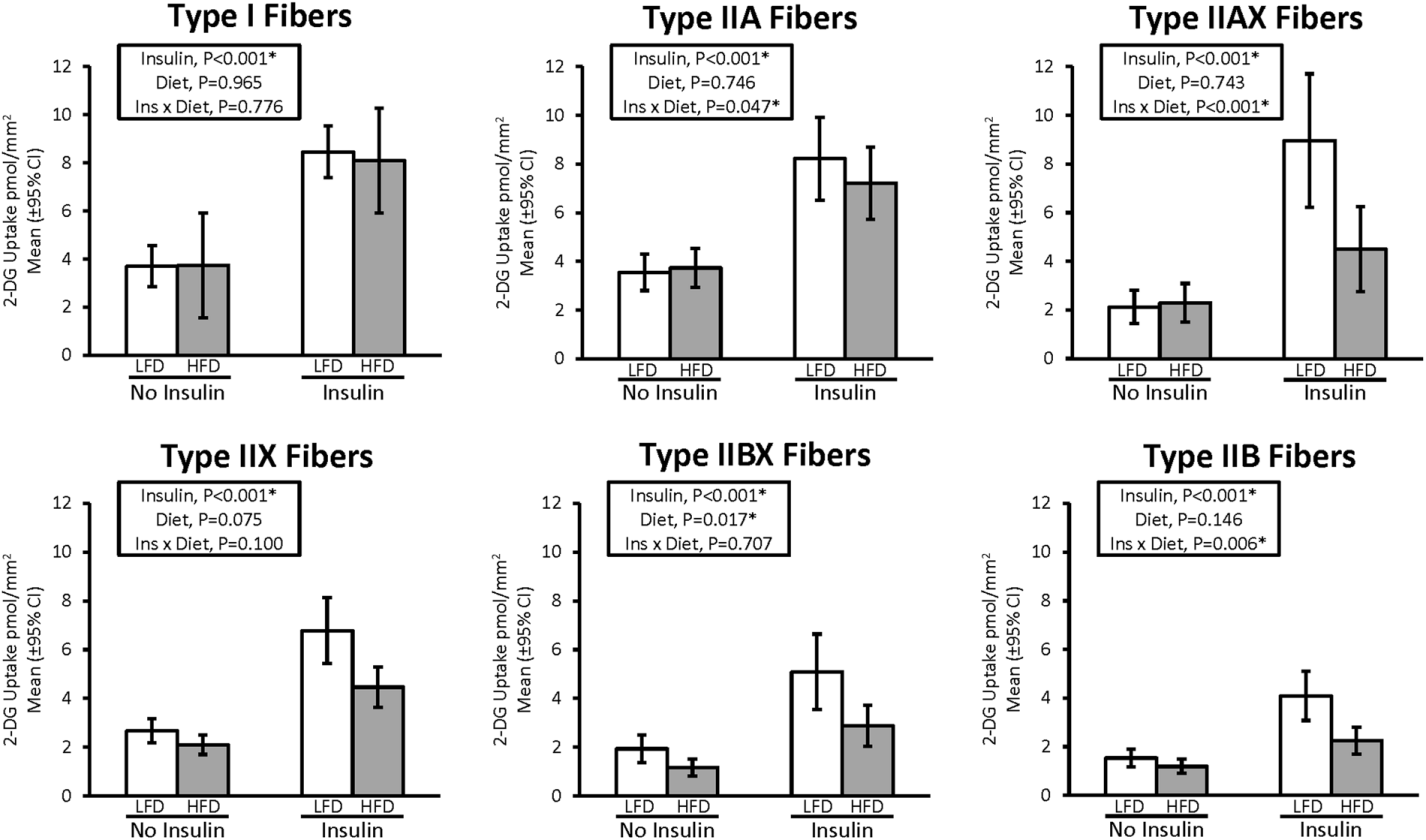
Effect of high fat diet (HFD) versus low fat diet (LFD) on 2-DG uptake in single fibers of each fiber type. P-values are displayed, and *P < 0.05 was considered statistically significant. Values are means ± 95% confidence interval.

All fiber types (I, IIA, IIAX, IIX, IIBX, and IIB) showed a significantly higher glucose uptake in the insulin treated muscles versus non-insulin-treated muscles (P < 0.001) (Fig. 2). We also observed a significant main effect of diet (P < 0.05) on glucose uptake in the type IIBX fibers, with higher uptake in the LFD relative to HFD. We observed an insulin by diet interaction effect on glucose uptake in type IIA (P < 0.05), IIAX (P < 0.001), and IIB fibers (P < 0.01), showing greater effects of diet within the insulin treated muscle relative to the non-insulin treated muscles. Additionally, values for type IIX fibers approached significance for a main effect of diet (P = 0.075) and an insulin and diet interaction (P = 0.100) for glucose uptake.

**Figure 3:**
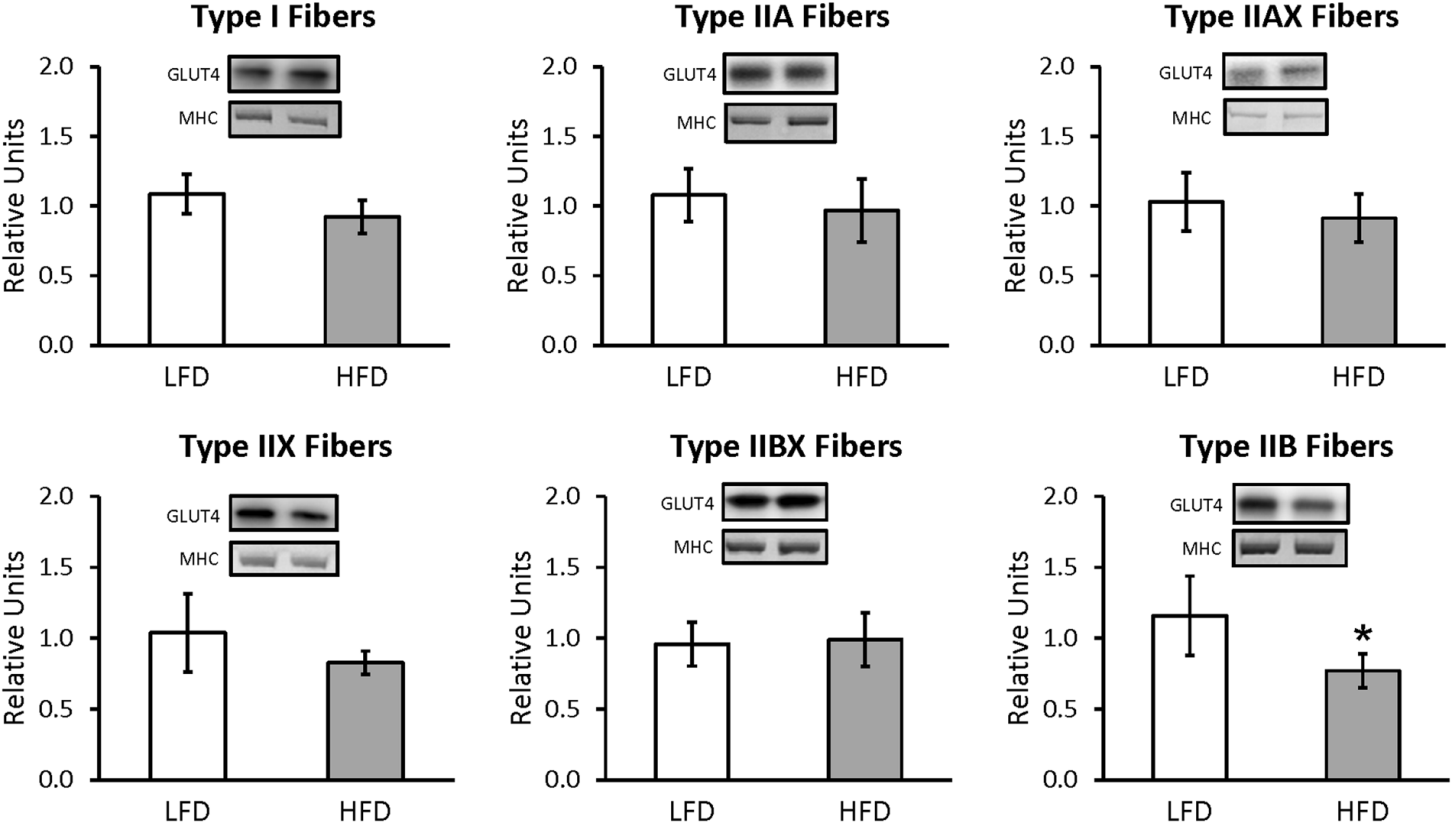
Effect of high fat diet (HFD) versus low fat diet (LFD) on the relative abundance of the glucose transporter protein, GLUT4, in single fibers of each fiber type. The loading control was myosin heavy chain (MHC). *Indicates a statistically significant difference between HFD and LFD (P < 0.05). Values are means ± 95% confidence interval.

**Figure 4:**
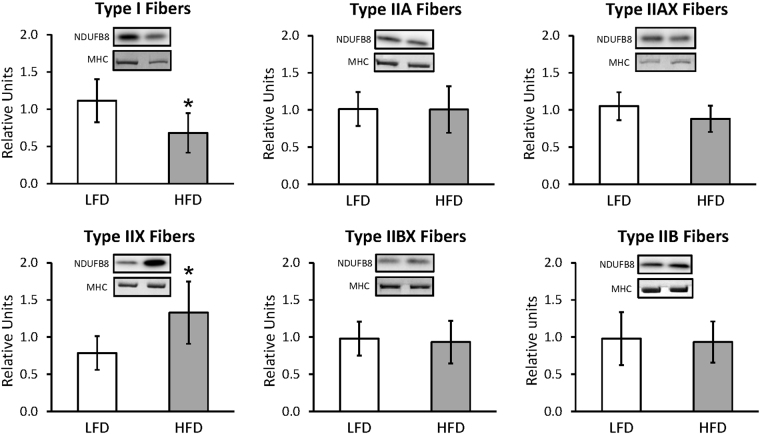
Effect of high fat diet (HFD) versus low fat diet (LFD) on the relative abundance of mitochondrial Complex I protein, NDUFB8, in single fibers of each fiber type. The loading control was myosin heavy chain (MHC). *Indicates a statistically significant difference between HFD and LFD (P < 0.05). Values are means ± 95% confidence interval.

**Figure 5:**
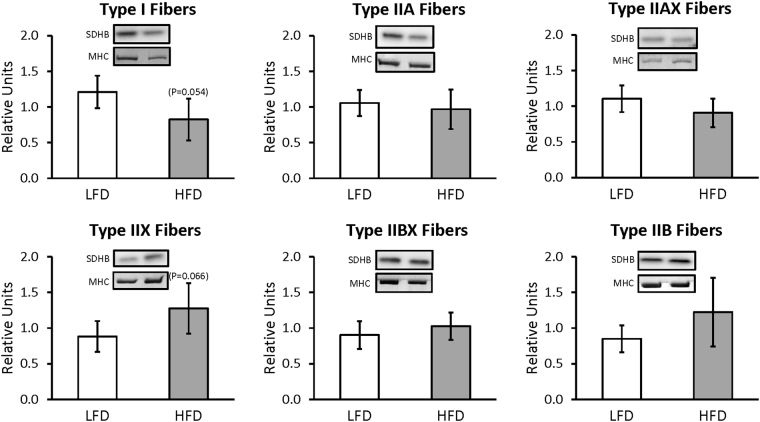
Effect of high fat diet (HFD) versus low fat diet (LFD) on the relative abundance of mitochondrial Complex II protein, SDHB, in single fibers of each fiber type. The loading control was myosin heavy chain (MHC). There was no significant difference between LFD and HFD for any fiber type. Values are means ± 95% confidence interval.

**Figure 6:**
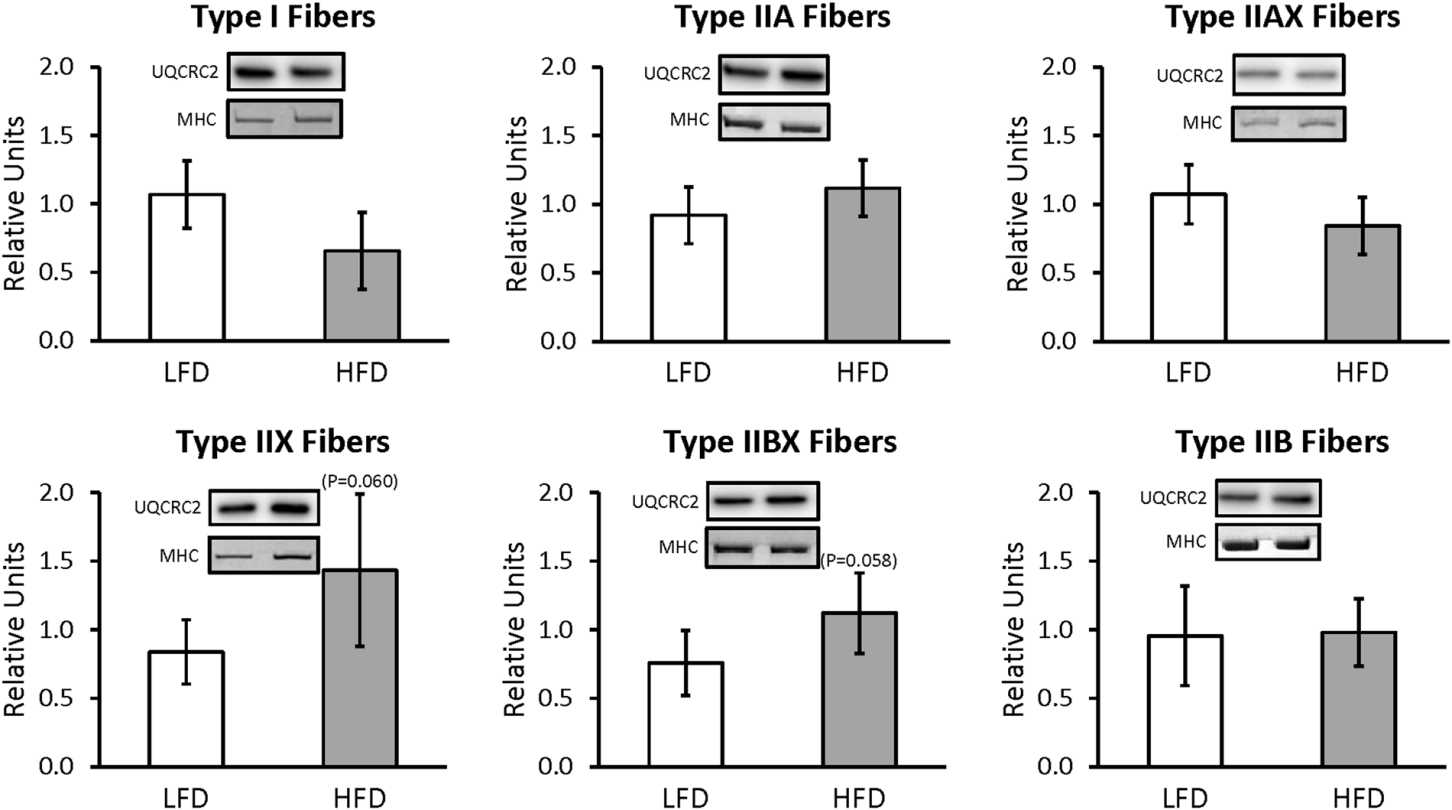
Effect of high fat diet (HFD) versus low fat diet (LFD) on the relative abundance of mitochondrial Complex III protein, UQCRC2, in single fibers of each fiber type. The loading control was myosin heavy chain (MHC). There were no significant differences between LFD and HFD for any fiber type. Values are means ± 95% confidence interval.

**Figure 7:**
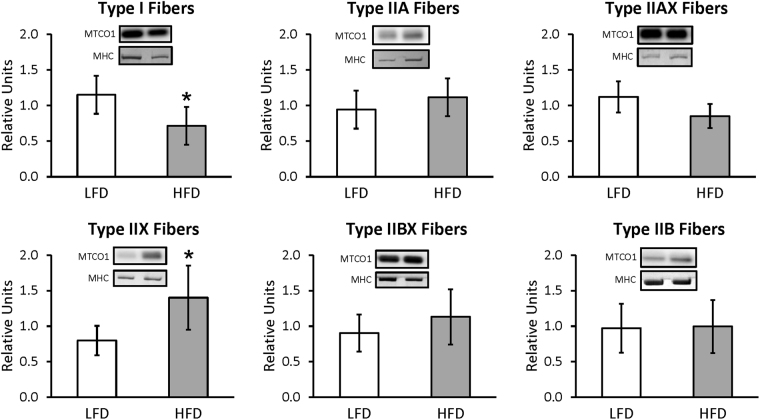
Effect of high fat diet (HFD) versus low fat diet (LFD) on the relative abundance of mitochondrial Complex IV protein, MTCO1, in single fibers of each fiber type. The loading control was myosin heavy chain (MHC). *Indicates a statistically significant difference between HFD and LFD at a level of P < 0.05. Values are means ± 95% confidence interval.

**Figure 8:**
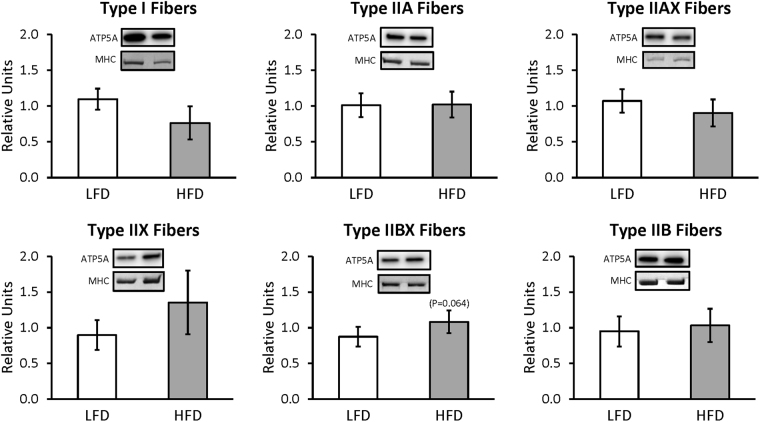
Effect of high fat diet (HFD) versus low fat diet (LFD) on the relative abundance of mitochondrial Complex V protein, ATP5A, in single fibers of each fiber type. The loading control was myosin heavy chain (MHC). There were no significant differences between LFD and HFD for any fiber type. Values are means ± 95% confidence interval.

**Figure 9:**
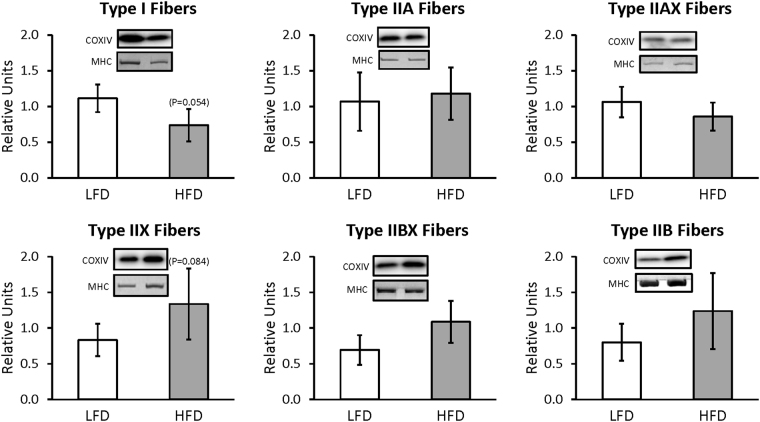
Effect of high fat diet (HFD) versus low fat diet (LFD) on the relative abundance of mitochondrial Complex IV protein, COXIV, in single fibers of each fiber type. The loading control was myosin heavy chain (MHC). There were no significant differences between LFD and HFD for any fiber type. Values are means ± 95% confidence interval.

For whole muscles from LFD versus HFD rats, no significant differences were detected for GLUT4 or any of the six mitochondrial proteins that were analyzed (NDUFB8, SDHB, UQCRC2, MTCO1, ATP5A, and COXIV) (Supplementary Figures 4 and 5). For single fibers, GLUT4 abundance was significantly decreased (P < 0.05) only in type IIB fibers of HFD versus LFD rats, with no significant difference in the other fiber types (Fig. 3). NDUFB8 protein abundance was significantly decreased (P < 0.05) in type I fibers and significantly increased (P < 0.05) in type IIX fibers from HFD compared to LFD rats (Fig. 4). In type I fibers from HFD compared to LFD rats, there was a non-significant trend for reduced abundance of SDHB (P = 0.054; Fig. 5). In type IIX fibers from HFD versus LFD rats, there was a non-significant trend for greater abundance of SDHB (P = 0.066; Fig. 5) and UQCRC2 (P = 0.06; Fig. 6). MTCO1 protein abundance was significantly decreased (P < 0.05) in type I fibers and significantly increased (P < 0.05) in type IIX fibers from HFD compared to LFD rats (Fig. 7). In type IIBX fibers from HFD compared to LFD rats, there was a non-significant trend for greater ATP5A abundance (P = 0.064; Fig. 8). In type I fibers from HFD compared to LFD rats, there was a non-significant trend for reduced abundance of COXIV (P = 0.054; Fig. 9). In type IIX fibers from HFD versus LFD rats, there was a non-significant trend for greater abundance of COXIV (P = 0.084; Fig. 9). Full length blots from single fiber analysis are displayed in the supplemental information (Supplementary Figure 6).

## Discussion

Assessing the insulin-stimulated glucose uptake of single muscle fibers that were individually characterized for their MHC isoform expression revealed novel insights at the cellular level that would have been imperceptible using only conventional muscle tissue analysis. The most important new results included: (1) a physiologic insulin dose significantly increased glucose uptake above basal values for each of the six fiber types analyzed (I, IIA, IIAX, IIB, IIBX and IIB) in both the LFD and HFD groups; (2) significant HFD-related decrements in glucose uptake (based on either main effect of diet or diet x insulin interaction) were identified for type IIA, IIAX, IIBX, and IIB fiber types along with a non-significant trend for an HFD-related decrease in IIX fibers and no evidence for HFD-related decrements for type I fibers; (3) the HFD-induced insulin resistance in whole muscle was attributable to fiber type-selective insulin resistance without any evidence for an alteration in the fiber type composition as determined by MHC isoform expression; (4) GLUT4 protein abundance was lower in type IIB fibers from HFD versus LFD rats, but not in whole muscles or in any of the other fiber types; and (5) HFD versus LFD rats differed with regard to abundance of several mitochondrial proteins in type I fibers (NDUFB8 and MTCO1 were greater for LFD) and type IIX fibers (NDUFB8 and MTCO1 were greater for HFD), but no significant differences were identified in whole muscles or in any of the other fiber types.

Because glucose uptake and MHC expression were both determined at the whole muscle and single fiber levels, it is possible to compare the magnitude of the directly measured HFD-induced insulin resistance for whole muscle with an estimate of the magnitude of whole muscle insulin resistance that relies on glucose uptake measured in single fibers. The relative abundance of each MHC isoform in whole muscle (8% type I, 16% type IIA, 27% type IIX, and 49% type IIB) can be multiplied by the respective decrements in glucose uptake of HFD versus LFD (non-significant 4% lower value for type I and significant decrements of 12% for type IIA, 34% for type IIX and 45% for type IIB). The sum of these isoform-selective values (33.5%) compares favorably to the 40% decrease that was directly measured in whole HFD versus LFD muscles. The similarity between these two values indicates that the single fiber results provide useful insights about whole muscle glucose uptake in addition to the insights at the cellular and fiber type-selective level that are uniquely revealed by single fiber analysis.

The current results identified fiber type-related differences in susceptibility to HFD-induced insulin resistance at the cellular level. Some^6,16,30^, but not all^13,14,31^ of the previous studies that evaluated muscle tissue glucose uptake using multiple muscles with differing fiber types have reported relatively greater insulin resistance for muscle enriched with type IIB fibers compared to muscles predominantly comprised of type I fibers. Careful scrutiny of the experimental designs and methods for the previously published whole muscle studies does not reveal a simple and obvious explanation for the differing results.

Multiple lines of evidence indicate that differences in muscle fiber type composition can influence insulin sensitivity. For example, whole body insulin sensitivity in humans was reported to be positively correlated to the percent of type I fibers in skeletal muscle^32^. In addition, insulin-stimulated glucose uptake is greater for rat skeletal muscle tissue enriched with type I and/or IIA fibers compared to muscles primarily comprised of type IIB fibers^33,34^. Furthermore, insulin-stimulated glucose uptake of single fibers expressing type I or IIA MHC exceeds the values for fibers expressing type IIB or IIX fibers^17–19^. Although a shift in fiber type composition would be predicted to influence insulin sensitivity, the HFD-induced insulin resistance in the whole epitrochlearis muscle in the current study was not accompanied by altered MHC isoform distribution. Rather, the current results demonstrate that the HFD-related insulin resistance in whole muscle was attributable to fiber type selective decrements in glucose uptake. It is notable that two earlier studies reporting fiber type changes in muscles of rodents eating a HFD found increased abundance of type I MHC rather than increased type IIB MHC^21,22^. Taking together these studies with the current results, the available evidence does not support the idea that altered fiber type composition is a major cause for HFD-induced insulin resistance.

Single fiber glucose uptake has been previously reported in only one other insulin resistant model, the obese Zucker (OZ) rat^17^. OZ rats have a mutation in the leptin receptor leading to extreme hyperphagia, and their excessive body fat is already evident at 2 to 3 wk-old^35^. Both the lean Zucker (LZ) and OZ rats in the earlier single fiber study were provided with ad libitum access to standard rat chow (i.e., LFD). Consistent with the typical obesity phenotype of OZ rats, it has been reported that ~8 wk-old OZ versus LZ rats have ~400% greater epididymal fat pad mass^36^ which greatly exceeds the increases observed in the current study (36% greater epididymal fat pad mass for HFD versus LFD). Single fibers from epitrochlearis muscles of LZ and OZ rats were stimulated using a supraphysiologic insulin dose (2000 µU/ml), and the relative magnitude of the genotype-related decrement (ranging from ~40 to 50%) in glucose uptake by insulin-stimulated fibers did not vary greatly among the four fiber types that were studied (IIA, IIB, IIX and IIB/X fibers)^17^. The substantially smaller change in glucose uptake of insulin-stimulated IIA fibers (16%) in the HFD rats from the current study compared to the ~40% decline in IIA fibers from OZ rats may be related to differences in the insulin resistance model and/or the insulin dose. Given the substantial differences between the OZ and brief HFD models with regard to their level of increased body fat and extent of insulin resistance for IIA fibers, it is striking that the relative decline in glucose uptake by IIB fibers from insulin-stimulated muscles after 2 wk of HFD (49%) was not less than the relative deficit in IIB fibers from OZ rats (41%). Insulin resistance is complex, so it should not be surprising that the extent of fiber type-related insulin resistance is not uniform across all insulin resistant conditions.

To gain insights into the potential mechanisms for the HFD-induced insulin resistance, we evaluated the GLUT4 content in whole muscles and single fibers. In the whole muscle, we confirmed our previous finding that a two-week HFD did not significantly alter total GLUT4 content of the epitrochlearis muscle^9^. The current study was apparently the first to determine the influence of HFD on both GLUT4 abundance and glucose uptake in single muscle fibers that had been characterized for fiber type. It seems reasonable to suspect that the lower abundance of GLUT4 protein found only in type IIB fibers contributed to the HFD-induced insulin resistance in type IIB fibers. However, the HFD-induced insulin resistance in other type II fiber types was not attributable to lower GLUT4 levels. Earlier research demonstrated a decline in insulin-stimulated GLUT4 localized in the cell surface membranes of whole epitrochlearis muscles from HFD compared to LFD rats^37^. We speculate that in the current study there was an HFD-induced decline in cell surface GLUT4 for each of the type II fiber types from insulin-stimulated muscles. Testing this idea will require the development of new methods that allow for the measurement of both cell surface GLUT4 and fiber type in the same fibers.

There is a great deal of interest, as well as controversy about the relationship between mitochondria and insulin sensitivity in skeletal muscle^26–29^. In whole skeletal muscles of rats consuming a HFD, the abundance of several mitochondrial proteins was previously reported to be unaltered after two weeks on the diet, but significant increases were evident for multiple mitochondrial proteins after four weeks^4^. The current study also found no detectable HFD effect on the abundance of six mitochondrial proteins in whole muscles after two weeks. However, single fiber analysis revealed compelling fiber type-selective HFD-effects on mitochondrial proteins that were obscured in whole muscle analysis. The lack of uniformity in the fiber type effects was evident even among the different type II fiber types that became insulin resistant, so these results do not point toward a simple relationship between changes in mitochondrial protein levels and induction of insulin resistance.

In conclusion, single fiber analysis revealed that the extent of HFD-induced insulin resistance can be profoundly variable for single muscle fibers expressing different MHC isoforms, even when the fibers are from the same muscles and rats. What might account for the striking fiber type-related difference in susceptibility to HFD-induced insulin resistance? In an earlier study^9^ analyzing whole epitrochlearis muscles using the same dietary protocols and the same insulin concentration as the current study, we found that the insulin-stimulated muscles from LFD rats exceeded HFD values for phosphorylation of Akt substrate of 160 kDa (AS160, also known as TBC1D4) on Thr^642^ and Ser^588^, two sites that are crucial for regulating insulin-stimulated GLUT4 translocation and glucose transport^38,39^. Our working hypothesis is that fiber type-selective deficits in insulin-stimulation of AS160 on Thr^642^ and Ser^588^ underlies the fiber type-selective insulin resistance that we revealed in the current study. We further predict that these fiber type-selective deficits in AS160 phosphorylation are responsible for fiber type-selective deficits in insulin-stimulated GLUT4 translocation. What might account for the putative fiber type-selective HFD-induced deficits in AS160 phosphorylation? At the whole muscle level, the same two week HFD protocol did not result in significantly diminished insulin-stimulated Akt activation^9^. However, it is conceivable that fiber type selective deficits in Akt signaling were obscured by whole tissue analysis, so analysis at the single fiber level would be appropriate. It is clear that further metabolic analysis at the single fiber level will provide uniquely valuable insights regarding the mechanisms underlying skeletal muscle insulin resistance.

## Methods

### Materials

The reagents and apparatus for SDS-PAGE and nonfat dry milk (no. 170–6404) were from Bio-Rad (Hercules, CA). [^3^H]-2-deoxyglucose (NET328001MC) and [^14^C] mannitol (NEC314250UC) were from PerkinElmer (Waltham, MA). Tissue Protein Extraction Reagent, T-PER (PI78510), Bicinchoninic Acid Protein Assay Kit (PI23223), MemCode Reversible Protein Stain Kit (PI24585), and SimplyBlue™ SafeStain (LC6065) were from ThermoFisher (Pittsburgh, PA). Collagenase type 2 (305 U/mg) was from Worthington Biochemical (LS004177, Lakewood, NJ).). Anti-rabbit IgG horseradish peroxidase conjugate (#7074) and anti-COXIV (#4850) were from Cell Signaling Technology (Danvers, MA). Anti-GLUT4 (#CBL243) was from EMD Millipore (Billerica, MA). Total Oxphos Antibody Cocktail (ab110413) was from Abcam (Cambridge, United Kingdom). The total OXPHOS Antibody Cocktail includes antibodies against five mitochondrial proteins involved in the electron transport chain and oxidative phosphorylation: NADH dehydrogenase (ubiquinone) 1β subcomplex subunit 8 (NDUFB8, part of Complex I); succinate dehydrogenase complex subunit 8 (SDHB, part of Complex II); ubiquinol-cytochrome-c reductase complex core protein 2 (UQCRC2, part of Complex III), Cytochrome c oxidase subunit I (MTCO1, part of Complex IV); and mitochondrial membrane ATP synthase (ATP5A, part of Complex V). Anti-mouse IgM (#sc-2973) horseradish peroxidase was from Santa Cruz Biotechnology (Santa Cruz, CA).

### Animal treatment

Procedures for animal care were approved by the University of Michigan Committee on Use and Care of Animals. All methods were performed in accordance with the guidelines from the Guide for the Care and Use of Laboratory Animals of the National Institutes of Health, USA. Male Wistar rats (6–7 weeks old; Charles River Laboratories, Boston, MA) were individually housed and provided with standard rodent chow (Laboratory Diet no. 5L0D; LabDiet, St. Louis, MO) or high-fat chow (Laboratory Diet no. D12492; ResearchDiets, New Brunswick, NJ) and water *ad libitum* for two weeks until they were fasted the night before the experiment at ~1700. Caloric intake for each rat during the two week diet period was estimated based on the difference between the food provided on day one and the food remaining at ~1700 on the night prior to the experiment. On the day of the experiment rats were anesthetized (intraperitoneal sodium pentobarbital, 50 mg/kg weight) at ~1000, weighed, and their epitrochlearis muscles were dissected. Muscles from 10 rats in each diet group were used for measuring glucose uptake and MHC abundance in whole muscles, and muscles from 10 rats in each diet group were used for measuring glucose uptake and fiber type in single fibers. After muscle dissections, the epididymal fat pads were dissected and weighed.

### *Ex vivo* incubations of muscles for single fiber and whole muscle glucose uptake

Dissected muscles used for single fiber glucose uptake were incubated in glass vials gassed (95% O_2_, 5% CO_2_) in a temperature controlled bath for a four-step process (35 °C during steps 1, 2 and 4; and step 3 was on ice) throughout all of the incubation steps. For step 1 (20 min) paired muscles were placed in vials containing 2 ml of media 1 (Krebs Henseleit Buffer, KHB, supplemented with 0.1% bovine serum albumin, BSA, 2 mM sodium pyruvate and 6 mM mannitol) with or without 100 µU/ml insulin. For step 2 (30 min), muscles were transferred to a vial containing 2 ml of media 2 [KHB supplemented with 0.1% BSA, 0.1 mM 2-DG (13.5 mCi/mmol [^3^H]-2-DG), 2 mM sodium pyruvate and 6 mM mannitol) with or without 100 µU/ml insulin. For step 3, muscles underwent three washes (5 min/wash with shaking at 115 revolutions/min) in ice-cold wash media (Ca^2+^-free KHB supplemented with 0.1% BSA and 8 mM glucose) to clear the extracellular space of [^3^H]-2-DG. For step 4 (60 min), muscles were incubated in vials containing collagenase media (wash media supplemented with 8 mM glucose and 2.5% type 2 collagenase) for enzymatic digestion of muscle collagen. Collagenase-treated muscles are hereafter referred to as fiber bundles.

Muscles used for whole muscle glucose uptake were incubated in glass vials gassed (95% O_2_, 5% CO_2_) in a temperature controlled bath for a two-step process (35 °C during both steps) throughout the incubation. These muscles were treated identically to muscles used for single fiber glucose uptake for incubation step 1. For incubation step 2 (30 min) these muscles were transferred to a vial containing 2 ml of media 3 [KHB supplemented with 0.1% BSA, 0.1 mM 2-DG (2.25 mCi/mmol [^3^H]-2-DG), 2 mM sodium pyruvate and 6 mM mannitol (2 mCi/mmol [^14^C] mannitol)] with or without 100 µU/ml insulin. After step 2, whole muscles were blotted, freeze clamped, and stored at −80 °C until further processing.

### Isolation and processing of single fibers for glucose uptake and MHC isoform identification

After incubation step 4, fiber bundles were removed from collagenase media, and rinsed with wash media at room temperature. Under a dissecting microscope (EZ4D; Leica, Buffalo Grove, IL), intact single fibers (~55 fibers per muscle) were gently teased away from the fiber bundle using forceps. After isolation, each fiber was imaged using a camera-enabled microscope with Leica Application Suite EZ software. After imaging, each fiber was transferred by pipette with 20 μl of wash media to a microcentrifuge tube. 30 µl of lysis buffer (T-PER supplemented with 1% Triton X-100, 1 mM Na_3_VO_4_, 1 mM EDTA, 1 mM EGTA, 2.5 mM sodium pyrophosphate tetrabasic decahydrate, 1 mM β-glycerophosphate, 1 μg/ml leupeptin, and 1 mM phenylmethylsulfonyl fluoride) and 50 µl of 2 × Laemmli buffer were added to each isolated fiber tube. Tubes were then vortexed and a portion of each lysed fiber was aliquoted into a separate tube for immunoblotting of GLUT4, MTCO1, and COXIV protein abundance, which are affected by heating. These samples were stored at −20 °C until used for immunoblotting. The remainder of the lysed fiber sample was heated to 95–100 °C for 10 min and was then stored at −20 °C until glucose uptake and MHC isoform abundance were determined.

### Single fiber glucose uptake

An aliquot (40 µl) from each lysed single fiber lysate was pipetted into a separate vial along with 8 ml of scintillation cocktail. The 2-[^3^H]-DG disintegrations per minute (dpm) in the aliquots from single fiber lysates together with the 2-[^3^H]-DG in the media (dpm per picomole) were then used to calculate each fiber’s accumulation of 2-[^3^H]-DG expressed relative to fiber area (picomoles x mm^−2^) that was determined based on images captured for each fiber as previously described^19^.

### Whole muscle glucose uptake

Frozen muscles used for GU were weighed and homogenized (Tissuelyser II homogenizer; Qiagen Inc., Valencia, CA) in ice-cold lysis buffer. Homogenates were then rotated at 4 °C for 1 h before centrifugation at 15,000 g for 10 minutes at 4 °C. Aliquots (200 µl) of supernatant were added to vials containing 8 ml of scintillation cocktail. 2-[^3^H]-DG and 2-[^14^C]-mannitol disintegrations per minute were measured by scintillation counter, and then 2-DG uptake was calculated as previously described^40^.

### MHC isoform identification

MHC isoforms in aliquots of single fiber lysates were separated and identified by SDS-PAGE essentially as previously described^19,41^. MHC isoform expression was determined by comparing the migration of MHC protein band(s) from each fiber or whole muscle homogenate with a MHC isoform standard [6 μg protein of a 3:2 mixture of homogenized rat extensor digitorum longus (EDL) and soleus muscles, E + S] containing all four MHC isoforms: I, IIA, IIB, and IIX.

### Immunoblotting

Total protein concentrations for whole muscle lysates were determined by bicinchoninic acid assay, and equal amounts of protein for each sample were separated via SDS-PAGE, and transferred to polyvinyl difluoride membranes. Aliquots of heated (95–100 °C) and non-heated single fiber lysates were separated by SDS-PAGE using 4–20% TGX gradient gels (#456–1096: Bio-Rad, Hercules, CA) or 10% gels, and then transferred to polyvinyl difluoride membranes. After electrotransfer, gels were stained in SimplyBlue™ SafeStain for 1 h at room temperature and then destained with deionized water for another 2 h. The SimplyBlue-stained MHC bands on the gels were quantified by densitometry (AlphaView; ProteinSimple, San Leandro, CA) and served as the loading controls for the subsequently immunoblotted proteins^42,43^. Membranes were incubated with appropriate concentrations of primary and secondary antibodies, and subjected to enhanced chemiluminescence (Luminata Forte Western HRP Substrate; #WBLUF0100; Millipore) to quantify protein bands by densitometry (FluoroChem E Imager, AlphaView software; ProteinSimple, San Leandro, CA). Individual values were normalized to the mean value of all samples on the membrane and divided by the corresponding MHC loading control value.

### Statistics

All data are expressed as mean ± 95% confidence interval (95% CI), with two-tailed significance levels of α < 0.05. Two-tailed *t*-tests were used to compare LFD and HFD groups for body weight, fat pad weight, caloric intake, fat mass to body mass ratio, MHC abundance (n = 10 for each diet group), and whole muscle protein abundance (n = 9–10 for each diet group). A two-way ANOVA was used to determine the effect of insulin and diet for whole muscle glucose uptake (n = 10 per group). Because single fiber glucose uptake and protein abundance data was collected from multiple individual fibers per rat, we evaluated these data using mixed-effects linear regression models, incorporating fixed parameters evaluating the contributions of diet (LFD, HFD) and insulin (insulin, no insulin) and their interaction effects, a random Y-intercepts to account for multiple observations within each animal. The analyses were performed using StataIC 14.2 (College Station, TX).

### Data availability

The datasets generated during the current study are available from the corresponding author on reasonable request.

## Electronic supplementary material

Supplementary Information
