# Supplementary material for: High-Fat Diet-Induced Insulin Resistance in Single Skeletal Muscle Fibers is Fiber Type Selective
Source: Sci Rep. 2017 Oct 20;7:13642. doi: 10.1038/s41598-017-12682-z (PMC5651812; doi:10.1038/s41598-017-12682-z)

High-Fat Diet-Induced Insulin Resistance in Single Skeletal Muscle Fibers is Fiber Type Selective

Mark W. Pataky, Haiyan Wang, Carmen S. Yu, Edward B. Arias, Robert J. Ploutz-Snyder, Xiaohua Zheng, and Gregory D. Cartee

Supplementary

Supplementary Figure 1. *Uncropped representative SDS-PAGE gel of whole muscle lysates.* Pooled rat extensor digitorum longus and soleus (E+S) standard was used to identify MHC isoform abundance in whole epitrochlearis muscle samples from LFD (low fat diet) and HFD (high fat diet) fed rats as is quantitatively reported in Figure 1.

Supplementary Figures 2 and 3. *Uncropped SDS-PAGE gels of single fiber lysates.* Representative gels display MHC isoform separation in individual muscle fibers to identify fiber type. Pooled rat extensor digitorum longus and soleus (E+S) standard was used to identify MHC isoform expression.

Supplementary Figure 4. *Immunoblots of GLUT4 and mitochondrial proteins from whole epitrochlearis muscles of LFD and HFD fed rats.* Representative blots together with the corresponding image for MHC which was used as a loading control. There were no significant differences between LFD and HFD groups for the abundance of any of these proteins.

Supplementary Figure 5. *Quantification of whole muscle immunoblots of GLUT4 and mitochondrial proteins.* Values are expressed relative to MHC loading control. There were no significant differences between LFD and HFD groups for the abundance of any of these proteins.

Supplementary Figure 6. *Immunoblots of GLUT4 and mitochondrial proteins from single muscle fibers of LFD and HFD fed rats.* Representative blots together with the corresponding image for MHC which was used as a loading control.

Supplementary Figure 1

Whole Muscle Gel

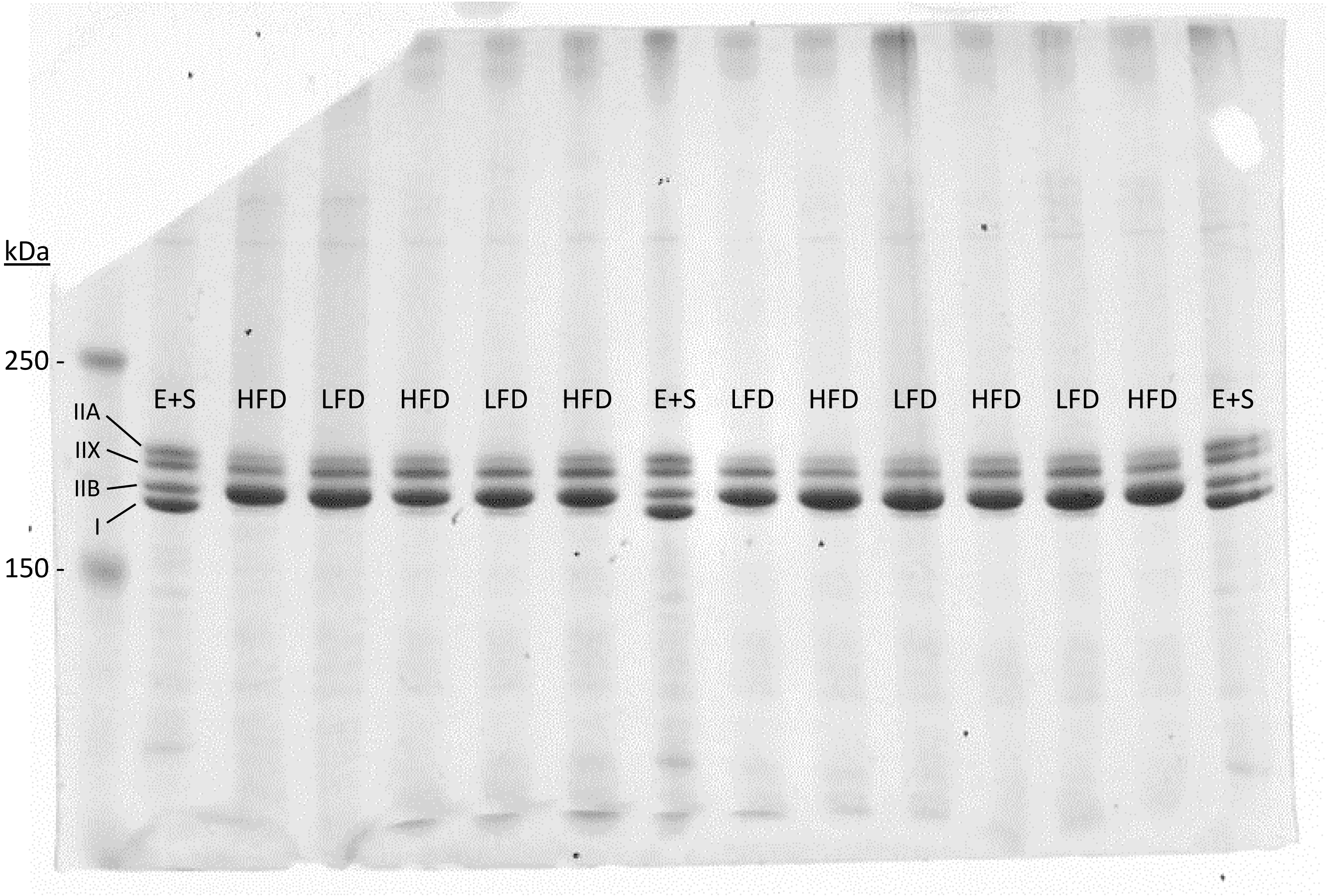

Supplementary Figure 2

Single Fiber Gel #1

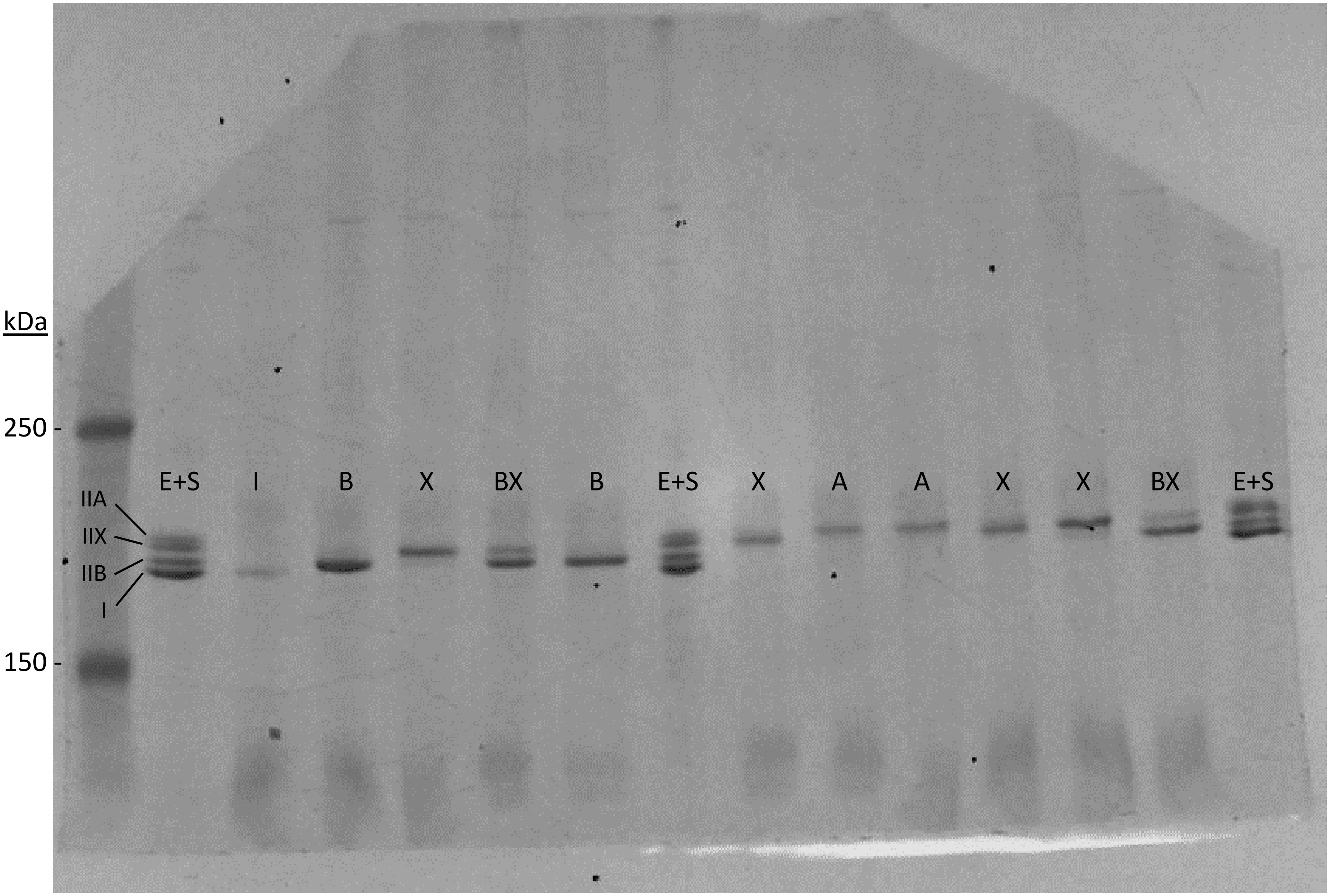

Supplementary Figure 3

Single Fiber Gel #2

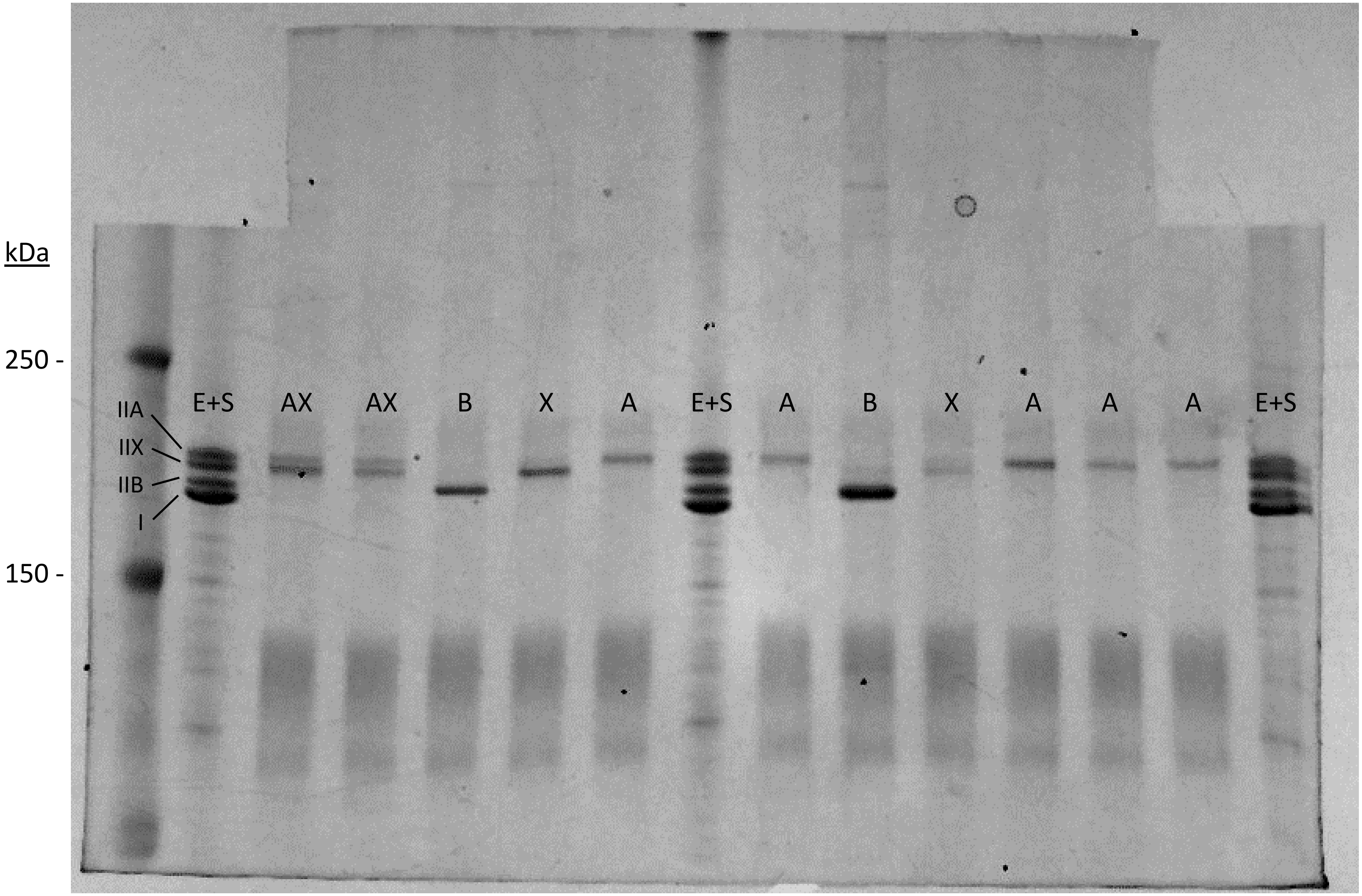

Supplementary Figure 4

Whole Muscle Immunoblot

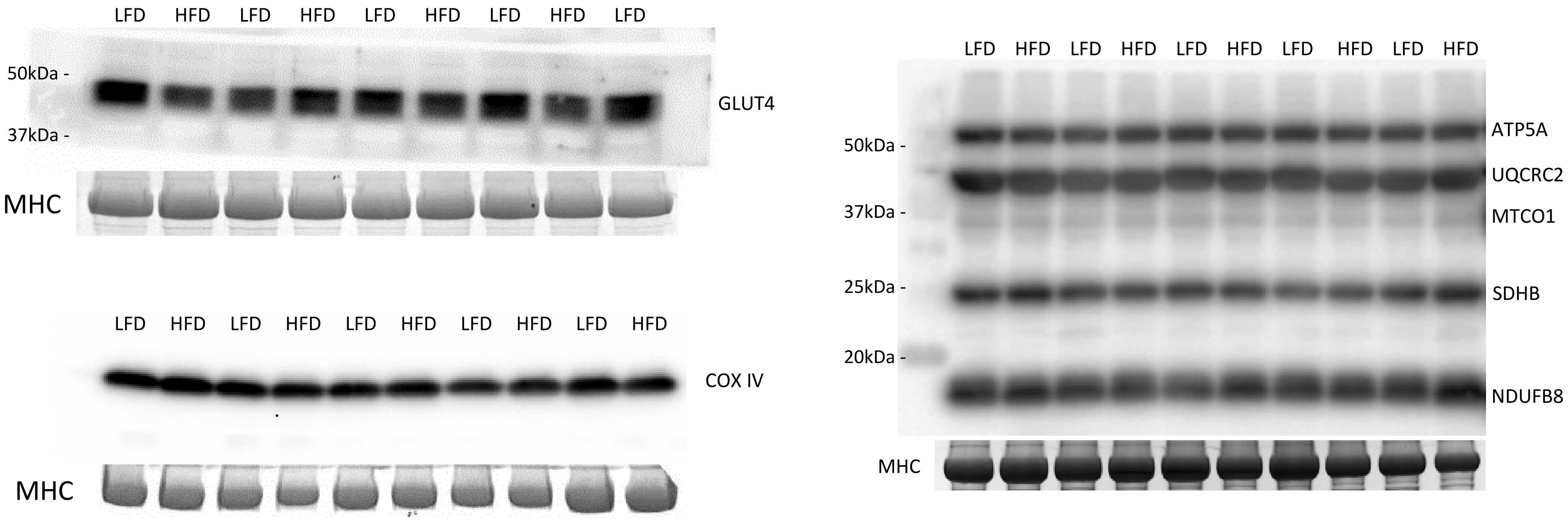

Supplementary Figure 5

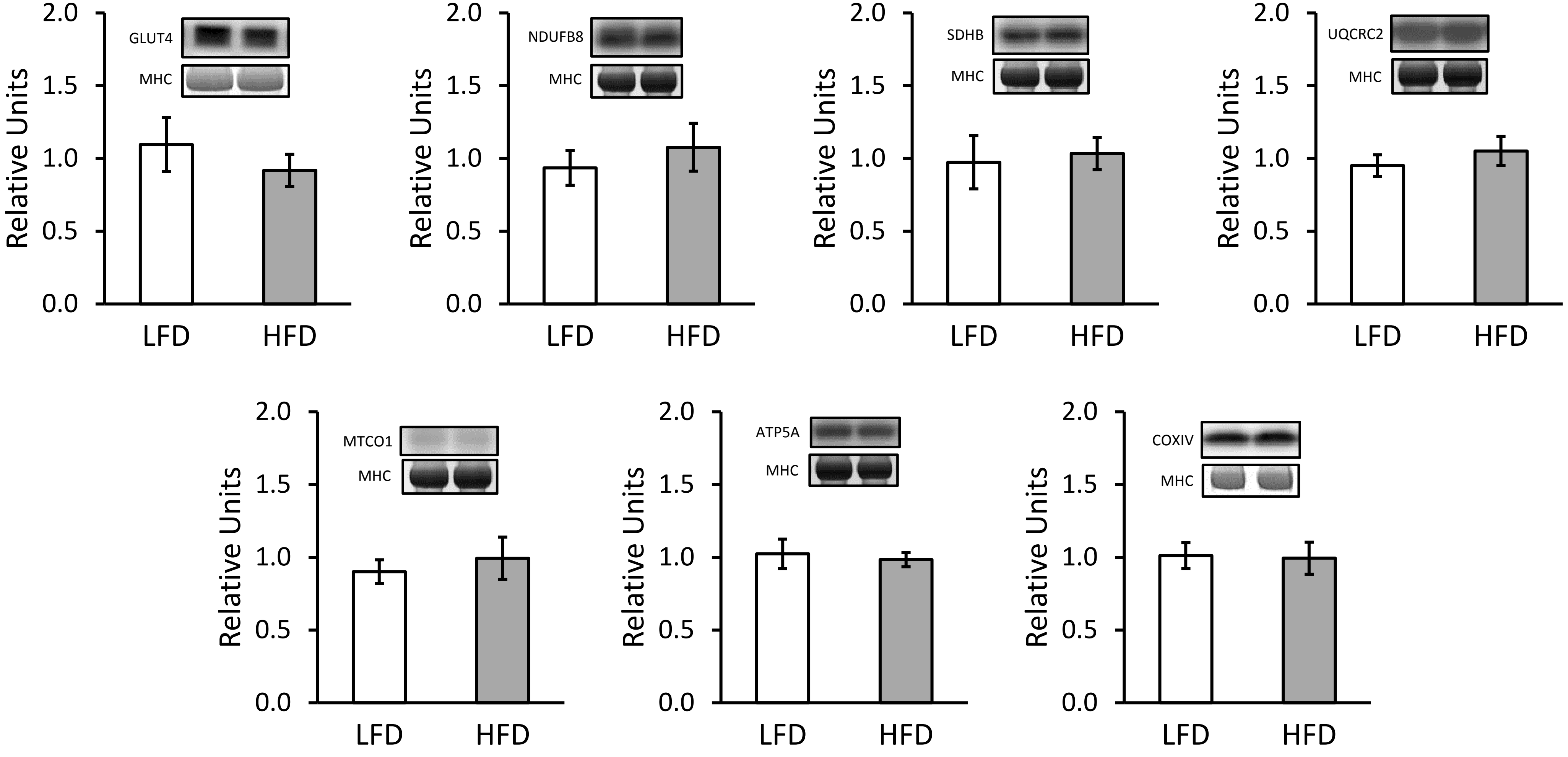

Supplementary Figure 6 <u>Single Fiber Immunoblot</u>

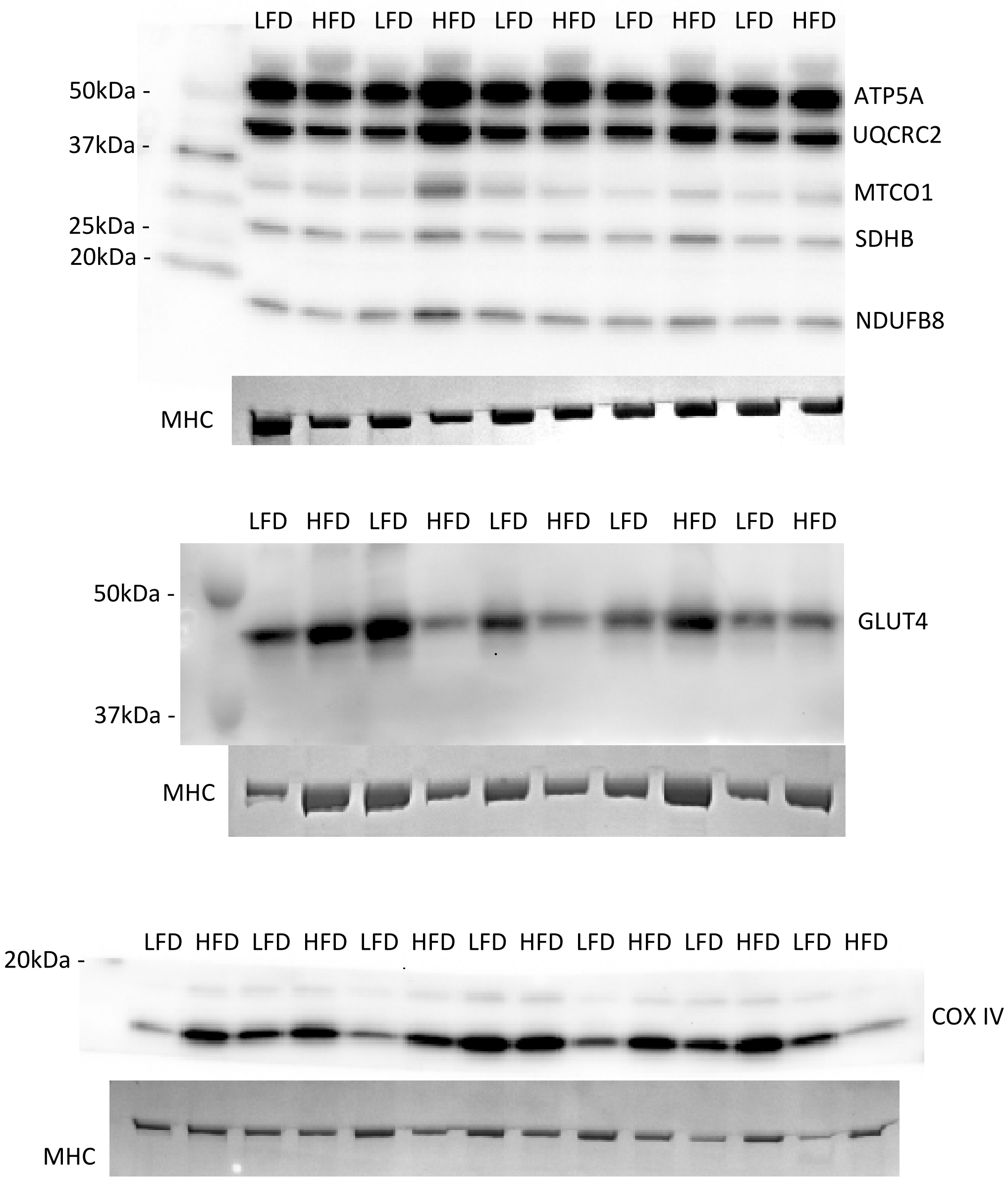

Supplement: Supplementary file 1 — Supplementary Information [file 41598_2017_12682_MOESM1_ESM.pdf]
